# Detection of hepatitis E virus RNA in processed pork products: a one-year prevalence study in Spain

**DOI:** 10.3389/fmicb.2026.1878664

**Published:** 2026-07-09

**Authors:** Mariana Alves Elois, Rafael Dorighello Cadamuro, Nadine Yeramian, Daniel Perez-Alonso, Antonio Valero Díaz, Rebeca Salguero Campomar, Henrique Borges da Silva Grisard, Álvaro Cañete Reyes, Gislaine Fongaro, David Rodríguez-Lázaro

**Affiliations:** 1 Laboratory of Microbiology, Department of Biotechnology and Food Science, University of Burgos, Burgos, Spain; 2 Centre for Emerging Pathogens and Global Health, University of Burgos, Burgos, Spain; 3 Laboratory of Applied Virology, Department of Microbiology, Immunology, and Parasitology, Federal University of Santa Catarina, Florianópolis, Brazil; 4 Department of Food Science and Technology, UIC Zoonosis y Enfermedades Emergentes (ENZOEM), University of Cordoba, Cordoba, Spain

**Keywords:** detection, food safety, hepatitis E, monitoring, pork meat

## Abstract

Hepatitis E virus (HEV) is an emerging zoonotic pathogen of global public health concern. The consumption of raw or undercooked pork products has been identified as a key route of transmission. In this study, the prevalence of HEV RNA was evaluated in commercially available processed pork products in Spain. A total of 280 samples were collected from retail stores and analyzed using a validated RT-qPCR. HEV RNA was detected in 5 samples, corresponding to an overall prevalence of 1.8% (95% CI: 0.6%–4.1%). All positive samples were identified in the first sampling quarter, with a significantly higher prevalence (8.9%, *p* = 0.001), while no detections were observed in subsequent quarters. No statistically significant associations were found between HEV detection and the presence of preservatives or product type. Among positive samples, viral loads were low, with a median of 12.8 copies/g and Ct values indicative of low-level contamination. HEV RNA was detected in several product categories, including chorizo, fuet, salchichón, and other processed meats. Overall, the results indicate that HEV RNA was detected at low prevalence and low viral loads in processed pork products, suggesting low-level molecular contamination. Although infectivity was not assessed, these findings remain relevant for molecular surveillance and may indicate a potential route of exposure through pork products.

## 1 Introduction

Hepatitis E virus (HEV) is an emerging zoonotic agent responsible for hepatitis E, a disease that represents a major global public health problem. It is estimated that HEV causes approximately 20 million infections annually, resulting in 3.3 million symptomatic cases and approximately 44,000 deaths (WHO, 2017).

HEV is a small, icosahedral, single-stranded, positive-sense RNA virus, approximately 7.2 kb in size and between 27 and 34 nm in diameter (Reyes et al., 1990). It belongs to the genus *Orthohepevirus* of the family *Hepeviridae*, which comprises the subfamilies *Orthohepevirinae* and *Parahepevirinae*. The *Orthohepevirinae* subfamily includes *Paslahepevirus* (HEV-A) and *Rocahepevirus* (HEV-C), which infect humans and domestic and wild mammals, *Chirohepevirus* (HEV-D), which infects bats, and *Avihepevirus* (HEV-B), which infects birds. The *Parahepevirinae* subfamily comprises only the genus *Piscihepevirus*, which contains *Paslahepevirus balayani* and *Paslahepevirus alci* (specific to moose) species (Purdy et al., 2022).

*Paslahepevirus balayani* (previously classified as *Orthohepevirus* A) infects humans and a variety of mammals. Among the eight recognized genotypes (HEV-1 to HEV-8), only genotypes 1–4 are associated with human infection. Genotypes 1 and 2 are primarily linked to epidemics in tropical and subtropical regions and are mainly transmitted via the fecal–oral route. In contrast, genotypes 3 and 4 are zoonotic and are responsible for sporadic cases or localized outbreaks in high-income countries, often associated with the consumption of raw or undercooked pork products (Ricci et al., 2017; Aslan and Balaban, 2020).

Pork is the second most consumed meat worldwide, with a global pig population estimated at 148.7 million animals in 2015, approximately 1.7 times higher than the cattle population (István and Vida, 2017).

Focusing on the European Union, the Spanish pork sector plays a pivotal role in the national economy, supported by one of the largest pig populations worldwide, with approximately 28.4 million animals. Spain is also the leading producer of pork in the European Union and ranks first in global exports (Mateos et al., 2024). In particular, the southwestern region of Spain is characterized by a strong market for Iberian pork, derived from a native breed of domestic pigs typically reared under extensive production systems, as well as for wild boar meat. Traditional pork products from this region, such as salchichón, chorizo, and cured ham are commonly consumed raw or undercooked (Bayarri et al., 2010; Martín et al., 2021; Tejada et al., 2021).

Domestic pigs are recognized as the main reservoir of HEV genotype 3. Studies conducted across several European countries have reported the detection of HEV genomic RNA in a wide range of meat products, including fresh meat, liver and organ products, various types of sausages (raw, fermented, and cured), and ready-to-eat processed foods (Moor et al., 2018; Bennett et al., 2024; López-López et al., 2024).

Beyond the detection of viral genetic material, HEV-related outbreaks have been reported following the consumption of roast suckling pig, roasted or grilled wild boar meat, and cured pork liver sausages in France, Italy, and Spain (Garbuglia et al., 2015; Guillois et al., 2016; Rivero-Juarez et al., 2017). In response to these concerns, the European Food Safety Authority (EFSA) has emphasized the need for integrated studies to assess the risk of transmission of HEV within the European food chain (Ricci et al., 2017).

In light of these considerations, the present study aimed to evaluate the prevalence of HEV RNA in commercially available pork products subjected to different processing methods intended for human consumption.

## 2 Materials and methods

### 2.1 Sampling strategy

Processed pork products were purchased quarterly from local retail stores in Burgos, Spain, between December 2024 and December 2025. Sampling was conducted in December 2024 and in March, June, September, and December 2025. Each sampling batch included 56 samples, resulting in a total of 280 samples analyzed. After purchase, samples were transported to the laboratory under refrigerated conditions, stored at 4 °C, and processed within 12 h.

The analyzed products included traditional Spanish processed pork products, such as chorizo, fuet, salchichón, longaniza, chistorra, blood sausage, pork loin products, puchero-related products, and Asturian compango. Based on the product names and ingredient lists, none of the analyzed products contained pig liver or liver-derived ingredients. For descriptive purposes, products were broadly classified according to processing characteristics as dry-cured or fermented sausages, including chorizo, fuet, salchichón, and longaniza; dry-cured whole-muscle pork products, including pork loin and related Iberian loin products; blood-based products, including blood sausage and achorizada; and fresh or semi-cured products generally intended to be cooked before consumption, including chistorra, puchero, and Asturian compango.

### 2.2 Internal process control virus

An internal process control virus (IPCV) was added to each sample immediately before the start of virus concentration and nucleic acid extraction. The IPCV used was murine norovirus 1 (MNV-1), propagated in RAW 264.7 cells to a concentration of 10^7^ TCID₅₀/mL. A spike containing approximately 3 × 10^3^ TCID₅₀ was added to each sample (Diez-Valcarce et al., 2011a, 2011b).

### 2.3 Virus concentration and nucleic acid extraction

Virus concentration was performed according to Santamaría-Palacios et al. (2025) with slight modifications. Briefly, a total of 300 mg of homogenized meat was placed into a 2-mL FastPrep tube containing 200 μL of phosphate-buffered saline and 2 g of 1-mm zirconia beads. Samples were subjected to three mechanical disruption cycles using a FastPrep-24 5G instrument (MP Biomedicals, USA), each consisting of 40 s at 6 m/s with 300 s intervals between cycles. Subsequently, 1 mL of QIAzol reagent (Qiagen, Germany) was added, and the mixture was vortexed for 30 s and incubated at room temperature for 5 min. Next, 200 μL of a 24:1 chloroform: isoamyl alcohol solution (Sigma-Aldrich) was added, followed by vortexing for 30 s and incubation at room temperature for 5 min. The tubes were centrifuged at 12,000 × *g* for 15 min at 4 °C, and the resulting supernatant was used for viral RNA extraction.

Nucleic acid extraction was performed on 150 μL of viral concentrate using the RNeasy Lipid Tissue Mini Kit (Qiagen, Hilden, Germany), which includes the QIAzol reagent, according to the manufacturer’s instructions and eluted in 60 μL of nuclease-free water. Three process controls were included in each analytical batch: (1) the negative process control consisted of 200 mL of autoclaved tap water processed as the samples, without MNV-1, and was used to monitor possible contamination; (2) the positive process control consisted of 200 mL of autoclaved tap water inoculated with MNV-1 and processed as the samples, to verify the efficiency of concentration, nucleic acid extraction, and quantification; (3) an extraction control was also included for each collection date by inoculating 3 mL of sterilized tap water, corresponding to the approximate final concentrate volume, with MNV-1. This control was subjected only to extraction and quantification to assess nucleic acid extraction efficiency (D’Agostino et al., 2011; Casado-Martín et al., 2025a).

### 2.4 Process control recovery

The control virus recovery was calculated by comparing the Ct value of the sample containing the control with the Ct value of the control alone, using the following formula:


$$\text{Recovery}(\%)=100\times 2^{\mathrm{Ct}\;\text{Control}-\mathrm{Ct}\;\text{Sample}}$$

Control virus recovery was classified as insufficient (<5%), acceptable (5%–25%), good (25%–50%), or very good (>50%). Values below 5% were considered unacceptable, and the virus concentration and nucleic acid extraction steps for the corresponding sample were repeated.

### 2.5 Detection of HEV by RT-qPCR

MNV-1 and HEV were quantified using a one-step RT-qPCR assay in a final reaction volume of 10 μL, containing 2.5 μL of RNA, 0.2 μM of primers, 0.2 μM of probe, and 1 × TaqMan Fast Virus 1-Step Master Mix (Applied Biosystems, USA). The primers and probe used for HEV detection were those previously described by Jothikumar et al. (2006), while the primers and probe used for MNV-1 detection were those described by Baert et al. (2008) and Jothikumar et al. (2006).

To assess the presence of potential RT-qPCR inhibitors, all samples were analyzed undiluted and at a 1:10 dilution, in duplicate. Potential matrix-related inhibition was evaluated by comparing amplification in undiluted and diluted extracts and by monitoring MNV-1 process control recovery. Improved amplification after dilution, delayed or absent amplification of the process control, or insufficient process control recovery were considered indicative of possible inhibition. Samples with unacceptable process control recovery were reprocessed to minimize the risk of false-negative results. For each virus and assay, a standard curve was generated using Quantitative Synthetic RNA from Hepatitis E virus (ATCC VR-3258SD).

RT-qPCR assays were performed using a QuantStudio™ 5 Real-Time PCR System (Applied Biosystems, USA), and data were analyzed with QuantStudio™ Design & Analysis Software (v1.5.1) (Applied Biosystems, USA). Thermal cycling conditions were adapted to the TaqMan Fast Virus 1-Step Master Mix protocol and consisted of reverse transcription at 50 °C for 15 min, initial denaturation at 95 °C for 2 min, followed by 45 amplification cycles at 95 °C for 15 s and 60 °C for 1 min.

Quantification was performed using a standard curve generated in each experiment to account for the intrinsic variability of RT-qPCR and to ensure reliable quantification. Samples were considered positive only when the cycle threshold (Ct) value was ≤40. A fixed fluorescence threshold was manually set based on software recommendations to ensure comparability across experiments (Casado-Martín et al., 2025b). Specifically, the threshold was set at 0.04 for HEV and 0.07 for MNV-1.

### 2.6 Study design and dataset

A retrospective observational study was conducted using a dataset of meat products analyzed for the presence of hepatitis E virus (HEV). The dataset comprised 280 individual samples, each corresponding to a single product unit. Samples were collected over five consecutive sampling periods (quarters) and classified according to product type and presence of preservatives.

For each sample, the outcome of HEV detection was recorded as a binary variable (positive/negative). For samples testing positive, additional data were available on cycle threshold (Ct) values and viral load expressed as copies per gram (copies/g).

### 2.7 Variables and data processing

The primary outcome variable was HEV detection, coded as 0 (negative) or 1 (positive). A secondary variable, viral quantification (copies/g), was available only for positive samples and was analyzed descriptively.

The main explanatory variables included:

- Quarter, treated as an ordered categorical variable to represent temporal progression (Quarter 1, Quarter 2, Quarter 3, Quarter 4, and Quarter 5, corresponding to December 2024, March 2025, June 2025, September 2025, and December 2025, respectively).
- Product group, obtained by grouping individual products into broader categories to reduce data sparsity (chorizo, fuet, salchichón, blood sausage, chistorra, and other products).
- Presence of preservatives, categorized as a binary variable according to the ingredient list (Yes or No).

Product types with low frequency were aggregated into a single category (“Other”) to ensure sufficient representation across groups and improve the stability of descriptive comparisons.

### 2.8 Descriptive analysis

Descriptive statistics were used to summarize the dataset. HEV prevalence (mean and 95% CI) was calculated as the proportion of positive samples overall and stratified by quarter, product group, and presence of preservatives.

Categorical variables were expressed as counts and percentages. Differences between groups were evaluated using Fisher’s exact test, given the low number of positive samples and the presence of small expected cell counts. A two-sided significance level of *α* = 0.05 was considered for all statistical tests.

For positive samples, Ct values and viral loads (copies/g) were summarized using median and range (minimum–maximum). No transformation or imputation was applied.

### 2.9 Statistical software

All analyses were performed using R software (R Foundation for Statistical Computing, Vienna, Austria). Data processing and descriptive analyses were conducted using base R and the tidyverse package.

## 3 Results

A total of 280 meat product samples were analyzed for the presence of HEV RNA (Table 1). Overall, 5 samples were positive, corresponding to an HEV detection prevalence of 1.8% (95% CI: 0.6%–4.1%). The mean virus recovery was 68.1%. Overall, 26.6% of the samples showed acceptable recovery (5%–25%), 12.8% good recovery (25%–50%), and 60.6% very good recovery (>50%).

**Table 1:** Characteristics of samples according to HEV detection.

Variable	Negative (*n* = 275)	Positive (*n* = 5)	Prevalence (95% CI)
Quarter			*p* = 0.001
1	51 (18.5%)	5 (100.0%)	8.9% (3.0%–19.6%)
2	56 (20.4%)	0 (0.0%)	0.0% (0.0%–6.4%)
3	56 (20.4%)	0 (0.0%)	0.0% (0.0%–6.4%)
4	56 (20.4%)	0 (0.0%)	0.0% (0.0%–6.4%)
5	56 (20.4%)	0 (0.0%)	0.0% (0.0%–6.4%)
Preservatives			*p* = 0.376
No	130 (47.3%)	1 (20.0%)	0.8% (0.0%–4.2%)
Yes	145 (52.7%)	4 (80.0%)	2.7% (0.7%–6.7%)
Product group			*p* = 0.925
Chorizo	117 (42.5%)	2 (40.0%)	1.7% (0.2%–5.9%)
Fuet	27 (9.8%)	1 (20.0%)	3.6% (0.1%–18.3%)
Salchichón	45 (16.4%)	1 (20.0%)	2.2% (0.1%–11.5%)
Other	45 (16.4%)	1 (20.0%)	2.2% (0.1%–11.5%)
Blood sausage	31 (11.3%)	0 (0.0%)	0.0% (0.0%–11.2%)
Chistorra	10 (3.6%)	0 (0.0%)	0.0% (0.0%–30.8%)

Data are presented as *n* (%). *p*-values were calculated using Fisher’s exact test. Prevalence is shown with exact 95% confidence intervals.

HEV-positive samples were detected exclusively in Quarter 1, in which 5 of 56 samples were positive, corresponding to a prevalence of 8.9% (95% CI: 3.0%–19.6%). No positive samples were identified in Quarters 2 to 5 (0.0, 95% CI: 0.0%–6.4% for each quarter). The distribution of detection across quarters was statistically significant (Fisher’s exact test, *p* = 0.001).

According to preservative use, 1 of 131 samples without preservatives was positive (0.8, 95% CI: 0.0%–4.2%), whereas 4 of 149 samples with preservatives were positive (2.7, 95% CI: 0.7%–6.7%). No statistically significant association was observed between preservative use and HEV detection (*p* = 0.376).

When grouped by product type, HEV-positive samples were identified in chorizo (2/119; 1.7, 95% CI: 0.2%–6.0%), fuet (1/28; 3.6, 95% CI: 0.1%–18.3%), salchichón (1/46; 2.2, 95% CI: 0.1%–11.5%), and other products (1/46; 2.2, 95% CI: 0.1%–11.5%). No positive samples were detected in blood sausage (0/31; 0.0, 95% CI: 0.0%–11.2%) or chistorra (0/10; 0.0, 95% CI: 0.0%–30.8%). However, the overall distribution of HEV detection according to product group was not statistically significant (*p* = 0.925).

Within Quarter 1, all samples were collected from the same retailer and during the same sampling date. The 56 Quarter 1 samples represented 29 commercial brands and six product groups: chorizo (*n* = 24), fuet (*n* = 6), salchichón (*n* = 8), blood sausage/morcilla (*n* = 7), chistorra (*n* = 2), and other products (*n* = 9). HEV RNA-positive samples were detected in chorizo (2/24), fuet (1/6), salchichón (1/8), and other products (1/9), whereas no positive samples were detected in blood sausage/morcilla (0/7) or chistorra (0/2). Brand-level stratification was performed for descriptive purposes only, because samples assigned to the same commercial brand did not necessarily represent the same product type or production batch. Consequently, commercial brand was used to describe the distribution of HEV RNA-positive samples within Quarter 1, but not to estimate brand-specific prevalence or infer differences in potential exposure risk between brands. Under this descriptive approach, the five HEV RNA-positive samples were distributed across five different anonymized brands, with no observed concentration of positive detections within a single commercial brand (Table 2).

**Table 2:** Brand-level distribution of HEV RNA-positive samples within Quarter 1.

Commercial brand category	Product-type distribution within brand/category, n	Samples analyzed, n	HEV-positive samples, n	HEV-positive product type
Brand A	Chorizo (*n* = 5)	5	1	Chorizo
Brand B	Fuet (*n* = 1)	3	1	Fuet
	Salchichón (*n* = 2)			
Brand C	Achorizada (*n* = 1)	1	1	Achorizada
Brand D	Chorizo (*n* = 1)	2	1	Chorizo
	Caña de Lomo (*n* = 1)			
Brand E	Chorizo (*n* = 1)	2	1	Salchichón
	Salchichón (*n* = 1)			
Other brands without HEV detection	Chorizo (*n* = 17)	43	0	—
	Fuet (*n* = 5)			
	Salchichón (*n* = 5)			
	Blood sausage/morcilla (*n* = 7)			
	Chistorra (*n* = 2)			
	Other products (*n* = 7)			
Total quarter 1	Chorizo (*n* = 24)	56	5	—
	Fuet (*n* = 6)			
	Salchichón (*n* = 8)			
	Blood sausage/morcilla (*n* = 7)			
	Chistorra (*n* = 2) Other products (*n* = 9)			

Commercial brands were anonymized. Brand A–E correspond to the five commercial brands in which HEV RNA-positive samples were detected.

### 3.1 Ct values and viral quantification in positive samples

Descriptive results for Ct values and viral loads among positive samples are presented in Table 3. The median Ct value was 38.5 (range: 35.0–39.3). The median viral load was 12.8 copies/g (range: 4.5–81.7 copies/g).

**Table 3:** HEV quantification and Ct values in positive samples.

Product	Product group	Ct value	Viral load (copies/g)
Chorizo	Chorizo	35.024	81.70
Chorizo	Chorizo	37.549	14.70
Fuet	Fuet	38.461	8.43
Achorizada	Other	38.651	12.80
Salchichón	Salchichón	39.314	4.50
Number of positive samples		Ct value, median (min-max)	Viral load (copies/g), median (min-max)
5		38.5 (35.0–39.3)	12.8 (4.5–81.7)

Only positive samples were included. Results are presented as median (min-max).

When stratified by product group (Table 4), the highest median viral load was observed in chorizo (48.2 copies/g, range: 14.7–81.7), followed by other products (12.8 copies/g), fuet (8.4 copies/g), and salchichón (4.5 copies/g).

**Table 4:** HEV quantification by product group.

Product group	*n*	Median copies/g	Range
Chorizo	2	48.2	14.7–81.7
Fuet	1	8.4	8.4–8.4
Salchichón	1	4.5	4.5–4.5
Other	1	12.8	12.8–12.8

Only positive samples were included.

Partial sequencing was attempted for all five HEV RNA-positive samples in order to obtain genotype information. However, no amplicons of sufficient quality/concentration for sequencing were obtained. This was likely related to the low amount of HEV RNA present in the positive samples, which showed high Ct values and low viral loads. Consequently, genotype assignment could not be performed.

## 4 Discussion

Overall, the present study indicates a low but detectable presence of HEV RNA in processed pork products. The positive samples were detected in chorizo, fuet, salchichón, and other processed pork products. Although the distribution across product groups was not statistically significant, HEV RNA detection in pork products not containing liver is generally reported at low prevalence, whereas higher positivity rates are more consistently observed in liver and liver-derived products.

A notable finding of this study was the temporal clustering of all HEV-positive samples in Quarter 1. One possible explanation is that the one-year prevalence period and quarterly sampling design may have provided only a limited temporal window to capture low-level and intermittent HEV RNA contamination in the retail pork chain. Boxman et al. (2020) reported differences in HEV RNA levels in raw pork sausages collected over multiple years, supporting the possibility that HEV RNA detection in pork products may vary over time.

This clustering may also be compatible with temporal variation at different stages of the pork production chain. Previous studies in swine and wild boar populations have described seasonal or production-stage-related variation in HEV infection (Rivero-Juarez et al., 2018). In addition, although pork slaughter and processing in Spain occur throughout the year, specific segments of the sector, particularly Iberian acorn-fed production, follow defined seasonal cycles, including an acorn-feeding period before slaughter and a regulated slaughter period between mid-December and late March. Therefore, seasonal variation in raw-material origin, slaughter timing, or processing flows may have contributed to the temporal clustering observed in this study (BOE, 2014).

To further explore the temporal clustering observed in Quarter 1, the available traceability-related information was examined. Because all samples were obtained from the same retail source by design, retailer-level clustering could not be evaluated. At the brand level, the five HEV RNA-positive samples belonged to five different anonymized commercial brands, with no observed concentration of positive detections within a single brand. However, because supplier, raw-material source, and production-batch information were not available for all products, the possibility of a common upstream source could not be confirmed or excluded.

Regarding preservative use, no statistically significant association was observed between HEV RNA detection and the presence of preservatives. However, since only five positive samples were identified, the statistical power to detect differences between groups was limited. Moreover, preservative status was classified according to the ingredient list, which does not capture preservative type or concentration, processing intensity, curing time, pH, water activity, or storage conditions. Therefore, the present results should not be interpreted as evidence that preservatives have no influence on HEV RNA persistence, but rather as an indication that no clear association was observed within this dataset.

An important limitation of the present study is that HEV detection was based on RT-qPCR, which should not be directly interpreted as evidence of infectious virus or as a quantitative estimate of consumer risk. This distinction is particularly important because the positive samples showed low viral loads and high Ct values, close to the assay’s analytical detection limit, indicating low-level HEV RNA contamination (Ricci et al., 2017; Locus et al., 2023). Consequently, these results should be interpreted as low-level molecular detections, with limited quantitative and biological inference regarding viral infectivity or consumer exposure risk. Nevertheless, the detection of HEV RNA in processed pork products remains relevant because these products may represent a potential route of exposure, especially when consumed without further heat treatment. EFSA has recognized HEV as a food-borne pathogen in Europe and identified raw or undercooked pork meat and liver as important sources of infection (Ricci et al., 2017), while other agencies have recommended that HEV be considered in hazard analysis and that surveillance data be collected along the pork production chain (ANSES, 2010). Therefore, molecular monitoring remains important for food-chain monitoring, risk assessment, and identification of potential contamination sources.

A further limitation of this study was the absence of genotype information for the HEV RNA-positive samples. Although partial sequencing was attempted, genotype assignment could not be achieved because the low viral loads and high Ct values of the positive samples did not yield amplicons suitable for sequencing. Therefore, it was not possible to determine whether the detected HEV RNA belonged to the genotype most associated with zoonotic foodborne transmission in Europe, or to compare these detections with human or swine HEV sequences circulating in Spain. Future studies should include molecular characterization of samples with sufficient viral loads, or employ more sensitive amplification and sequencing approaches, to enable phylogenetic comparison.

The low overall prevalence observed in the present study is consistent with previous studies reporting relatively low HEV RNA detection rates in similar non-liver pork matrices. Di Bartolo et al. (2012) reported a prevalence of 2% (6/313) in sausages, interestingly detected only in Spain, the same country where the present study was conducted (Di Bartolo et al., 2012).

Similarly, Berto et al. (2012) detected HEV RNA in 10% (6/63) of sausage samples collected in the United Kingdom (Berto et al., 2012). Interestingly, a recent study performed in Spain by López-López et al. (2024) also identified a low presence of HEV contamination in cured, salted and cured, or boiled pork products. In that work, the authors conducted a large-scale analysis of 1,265 pork products, obtaining a positivity rate of only 0.4% (López-López et al., 2024). Other studies have reported no HEV RNA detection in specific pork meat products or selected non-liver product categories (Markantonis et al., 2018; Pallerla et al., 2021; Soares et al., 2022; Locus et al., 2023).

In contrast, higher prevalence rates have also been reported in other contexts. In the Netherlands, Boxman et al. (2020) observed a positivity rate of 14.6% (46/316) in raw pork sausages, including products such as *cervelaat*, salami, *metworst*, and *snijworst*. However, HEV RNA was detected significantly more frequently in these raw pork sausages than in other types of raw pork products, such as dried sausages, fuet, or chorizo, which were also analyzed in that study (Boxman et al., 2020). In Germany, HEV RNA was detected in 20% of raw sausage samples (14/70) (Szabo et al., 2015), while in Belgium, HEV RNA was found in 15% of raw dried ham samples (2/13) (Locus et al., 2023).

These differences in HEV detection rates among studies may reflect variations in product type, processing conditions, and contamination of raw materials, all of which can influence the persistence and detectability of viral RNA. Although these are product- and food-chain-related factors, they also fit within a broader One Health framework. HEV circulation in pork products is not determined only by the characteristics of the final product, but also by the infection dynamics in animal reservoirs, the contamination status of raw materials, and the possible contribution of environmental sources within the production chain. In this context, Ahmad et al. (2022) emphasized the role of pigs as major HEV reservoirs and highlighted the relevance of livestock-, food-, water-, and environment-related pathways in HEV transmission (Ahmad et al., 2022). Therefore, differences in HEV detection across pork product types should be interpreted not only as a consequence of processing or matrix composition, but also as a reflection of upstream variability in viral circulation and contamination along the meat supply chain. This reinforces the importance of continued HEV monitoring from a One Health perspective.

## 5 Conclusion

Taken together, these findings indicate that HEV RNA can be detected at low prevalence in processed pork products. The absence of statistically significant associations with product type and preservative use should be interpreted cautiously, given the low number of positive samples and the limited statistical power to detect differences between groups. Therefore, these results do not exclude a possible influence of product characteristics, processing conditions, or raw material contamination on HEV RNA detection.

The temporal clustering of positive samples in Quarter 1 suggests possible temporal variability in HEV RNA contamination. However, the presence of HEV RNA does not provide information on viral infectivity and should not be interpreted as direct evidence of infectious viruses. Nevertheless, molecular surveillance of HEV remains important to better understand its occurrence in processed pork products, assess its public health significance, and identify potential sources of contamination along the food production chain.

## Data Availability

The raw data supporting the conclusions of this article will be made available by the authors, without undue reservation.
